# Distance to Care and Telehealth Abortion Demand After *Dobbs*

**DOI:** 10.1001/jamanetworkopen.2025.38212

**Published:** 2025-10-20

**Authors:** Amy K. Willerford, Emily M. Godfrey, Caitlin Myers, Rebecca Gomperts, Erin K. Thayer, Anna Fiastro

**Affiliations:** 1 University of Washington School of Medicine, Seattle; 2 Department of Family Medicine, University of Washington, Seattle; 3 Department of Economics, Middlebury College, Middlebury, Vermont; 4 Aid Access, Amsterdam, the Netherlands; 5 University of Southern California, Los Angeles

## Abstract

This cross-sectional study of public health data in 18 US states examines the change in telehealth medication abortion requests after the 2022 *Dobbs v Jackson Women’s Health Organization* Supreme Court decision.

## Introduction

Geographic distance has become an increasingly critical determinant of abortion access in the aftermath of the Supreme Court decision *Dobbs v Jackson Women’s Health Organization*.^1,2^ As distance increases, so do delays in care and the cost of accessing an abortion—factors that may eliminate access altogether.^2,3^ Many patients are turning to telehealth medication abortion (teleMAB) services, citing benefits including lower costs and eliminating travel.^2,4,5^

While the surge in teleMAB is well documented, the extent to which distance from a brick-and-mortar abortion clinic relates to this demand in states that permit abortion post-*Dobbs* remains unclear.^2,4,6^ We aim to characterize the changes in teleMAB requests before and after *Dobbs* and examine the association between request rates and distance to an abortion facility over the same period.

## Methods

We conducted a repeated cross-sectional study to examine Aid Access asynchronous teleMAB service requests before and after *Dobbs*, stratified by distance to the nearest abortion facility. We used data from 18 states where teleMAB was legal 8 months before and after *Dobbs* to ensure comparability across jurisdictions and to minimize legal and reporting variability.

We calculated the number of teleMAB requests per county and month and determined distance to the nearest abortion facility using population-weighted county centroids from the Myers Abortion Facility Database. We used Housing and Urban Development United States Postal Service (HUD-USPS) zip code and county name data to match the Federal Information Processing Standards (FIPS) codes associated with patient records.

We described trends in completed teleMAB requests over time and used Poisson models with a quadratic distance specification to estimate the association between distance to the nearest brick-and-mortar facility and county-level per capita teleMAB requests, including an interaction term for time. We analyzed data using R version 3.6.1 (R Project for Statistical Computing) and StataIC (Stata Inc); this study followed Strengthening the Reporting of Observational Studies in Epidemiology (STROBE) reporting guidelines. The University of Washington Human Subjects Division determined this study did not require review or informed consent because it did not involve human participants.

## Results

**Figure 1.**
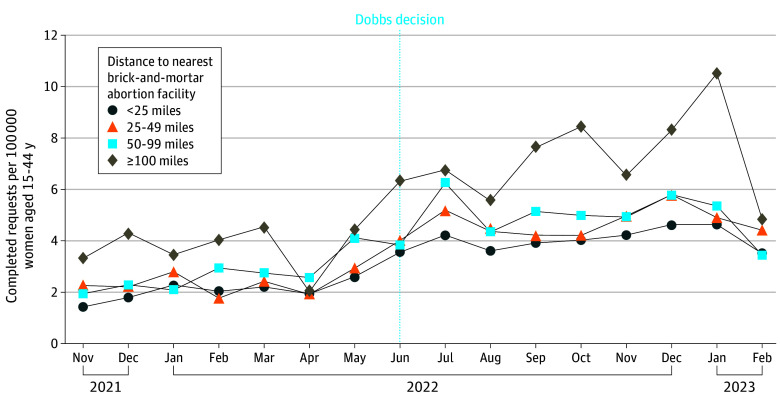
US County-Level Trends in Completed Telehealth Medication Abortion Service Requests by Distance to the Nearest Brick-And-Mortar Abortion Facility Vertical line indicates the *Dobbs v Jackson Women's Health* 2022 Supreme Court decision.

**Figure 2.**
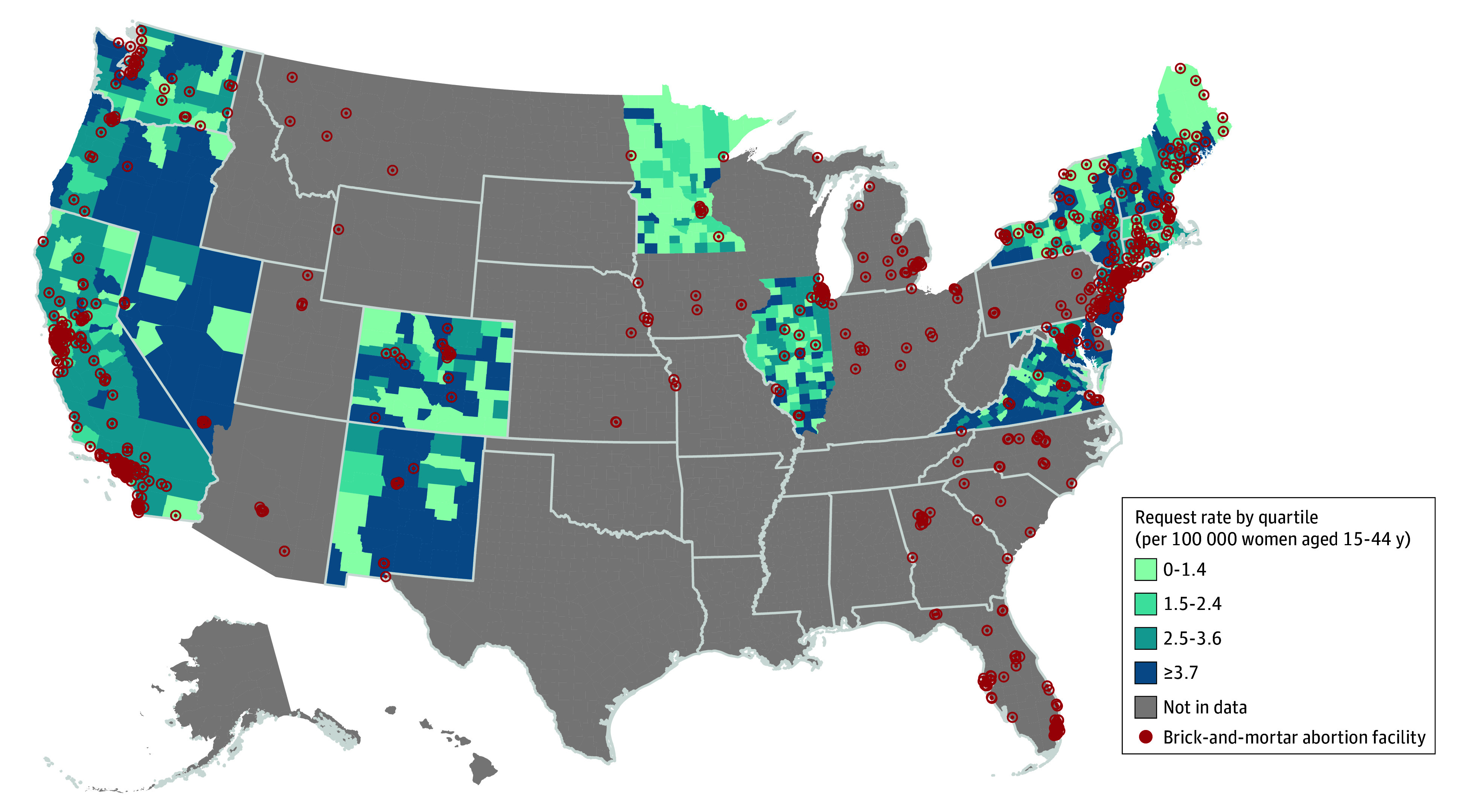
Average Monthly Completed Telehealth Medication Abortion Service Request Rate by US County Between November 2021 and February 2023 Red markers indicate the locations of brick-and-mortar abortion facilities as recorded in the Myers Abortion Facility Database.

Between November 2021 and February 2023, Aid Access completed 16 154 teleMAB requests across 18 states and 743 counties. Individuals had a median (IQR) age of 26 (22-31) years and most were less than 6 weeks pregnant (9557 [59.2%]) and had no children (9495 [58.8%]). There were 4545 requests pre-*Dobbs*, and 11 609 after. The average monthly teleMAB request rate rose across all counties post-*Dobbs*, from 2.4 to 4.5 requests per 100 000 women aged 15 to 44 years per month (Figure 1). On average, this rate increased the further individuals lived from brick-and-mortar facilities (Figure 2). The teleMAB request rate tended to be highest in counties located 100 miles or more from an abortion facility both before and after *Dobbs*. A 100-mile increase in distance was associated with a 13% increase in requests per capita (95% CI, 7% to 19%) pre-*Dobbs* and an 8% increase (95% CI, 3% to 13%) post-*Dobbs*; these increases are not statistically different from one another (−5.5%; 95% CI, −25.6% to 4.5%; *P* = .59).

## Discussion

Overall, we observed a doubling in monthly teleMAB requests across 18 states post-*Dobbs*, with most requests occurring before 6 weeks of pregnancy and the highest rates among individuals living further from in-person abortion care. These results build upon our findings pre-*Dobbs*, which also demonstrated increased teleMAB requests among individuals living further from brick-and-mortar facilities.^2^

An important study limitation is the reliance on data from states where abortion remained legal post-*Dobbs*, which may have included persons traveling from restricted states for care. Still, our findings align with growing evidence that teleMAB is essential for those living far from in-person care, and its availability enables individuals to receive treatment at early gestations, thereby reducing complications that can occur with delayed care.^4,6^

The loss of federal abortion protections has significantly disrupted access even in states with robust service provision.^1^ Our results highlight the critical role of teleMAB in reducing geographic barriers to care and underscore the need to expand teleMAB access nationwide.

## Notes

Supplement.
Data Sharing Statement
