# Supplementary material for: Distance to Care and Telehealth Abortion Demand After Dobbs
Source: JAMA Netw Open. 2025 Oct 20;8(10):e2538212. doi: 10.1001/jamanetworkopen.2025.38212 (PMC12538371; doi:10.1001/jamanetworkopen.2025.38212)
Supplement: Supplement. — Data Sharing Statement [file jamanetwopen-e2538212-s001.pdf]

Data Sharing Statement

Willerford. Distance to Care and Telehealth Abortion Demand After Dobbs. *JAMA Netw Open*. Published October 20, 2025. doi:10.1001/jamanetworkopen.2025.38212

Data
Data available: No

Additional Information
Explanation for why data not available: The data used in this study are not publicly available due to the sensitive nature of patient information and legal considerations surrounding abortion access.
